# Research on the Influence of Substrate Surface Roughness on the Sensing Performance of Fiber Bragg Gratings

**DOI:** 10.3390/s26051633

**Published:** 2026-03-05

**Authors:** Jiongyao Du, Yongxing Guo, Yongjian Gong, Chang Liu, Jian Lu

**Affiliations:** 1 Key Laboratory of Metallurgical Equipment and Control Technology, Ministry of Education, Wuhan University of Science and Technology, Wuhan 430081, China; dujyao@163.com (J.D.); yongjian629@wust.edu.cn (Y.G.); lc5350250@163.com (C.L.); lujian@wust.edu.cn (J.L.); 2 The State Key Laboratory of Advanced Refractories, Wuhan University of Science and Technology, Wuhan 430081, China

**Keywords:** Fiber Bragg Grating, substrate surface roughness, sensing characteristics

## Abstract

The packaging process of fiber Bragg gratings (FBGs) directly determines the strain transfer efficiency, chirp occurrence, and sensing performance of the sensors. At present, relevant theoretical and experimental studies on the surface roughness of packaging substrates remain scarce. In this paper, combined with the theoretical model of interfacial debonding driving force and the sensing mechanism of FBGs, the FBG sensing performance under different substrate surface roughness conditions was investigated. Experimental results show that an excessively high substrate surface roughness will induce FBG chirp when the external strain reaches 1.143 × 10^−3^, leading to the failure of strain transfer. In contrast, an excessively low surface roughness will weaken the interfacial coupling, thus reducing the strain response capability and cyclic stability of the sensor. The substrate surface treated by sandblasting with 150# abrasive exhibits the optimal comprehensive performance: The strain response capability of the FBG reaches 6.99994 × 10^−6^ pm/ε with a linear fitting coefficient of 0.99994, presenting excellent linear response and cyclic stability. This study clarifies the optimal range of substrate surface roughness for FBG packaging and can provide theoretical and technical references for the packaging design and optimization of high-performance FBG sensors.

## 1. Introduction

Fiber Bragg grating (FBG) packaging technology serves as a critical bridge linking fundamental research to engineering applications and is essential to advancing the practical implementation of FBG sensing technology. Effective packaging is therefore key to ensuring the performance and durability of the sensors, enabling them to operate accurately across diverse application scenarios [1,2,3]. When external loads such as pressure and strain act on the packaging substrate, the strain generated in the substrate is efficiently transferred to the grating region via interfacial coupling. This induces regular variations in the effective refractive index and period of the grating, which are ultimately manifested as shifts in the reflected wavelength for signal output, thereby achieving precise quantification of the external loads. By employing appropriate substrate materials, optimizing structural design, and refining interfacial treatment processes, high-quality packaging can provide robust support and reliable protection for the FBG sensing element, effectively attenuating external interference and suppressing signal attenuation and distortion. Such advances are of great practical significance in promoting the deployment of FBG sensing technology in fields including aerospace, infrastructure, petroleum and natural gas, and construction [4,5,6]. Accordingly, numerous scholars worldwide have conducted extensive explorations and experimental attempts regarding FBG packaging technology.

Some scholars have encapsulated FBGs with heat-sealing tape [7] and heat-sealed silicone tubing [8], which can provide consistent and reliable measurements under dynamic conditions while minimizing signal interference. Other researchers have employed novel composite materials for FBG encapsulation, such as β-eucryptite [9], glass frits [10,11], composites made of Terfenol-D particles and epoxy resin [12], acrylic gel [13], carbon fiber-reinforced polymer (CFRP) [14], and polydimethylsiloxane (PDMS) [15]. Additionally, laser welding techniques with special solders [16] have also been adopted for FBG encapsulation. Experimental results have verified that all these approaches can enhance the resistance of FBG sensors to environmental corrosion, ensure their long-term stability, and achieve a substantial improvement in sensing sensitivity.

Other scholars have adopted 3D printers to realize the design of novel substrate structures [17] or fabricated substrates using advanced materials [18], and the prepared sensors exhibit significant improvements in sensitivity and other performance indicators. Other researchers have encapsulated FBGs in capillaries [19,20,21], which reduces the overall dimensions of the sensors and expands their applicable scenarios. Furthermore, the sensing substrate is selected according to specific application scenarios. Substrates such as ceramic ferrules [22], silicon-on-insulator (SOI) platforms [23], thick-walled metal cylinders [24], and pre-bent elastic curved beams [25] enable the sensors to achieve rapid and reliable response as well as high sensitivity under particular working conditions.

The measurement sensitivity, linear response characteristics, and long-term stability of sensors constitute the core technical indicators that determine the engineering application of FBG sensing systems. Scholars at home and abroad have conducted extensive exploratory research on the packaging structure design and packaging materials of FBG, proposing a series of distinctive packaging schemes. However, most of the existing research focuses on the mechanical transmission characteristics of the packaging structure or the innovation of packaging materials, while there is relatively little research on the influence of the key interface parameter of the encapsulation substrate surface roughness on the sensing performance of FBG. The correlation law between the surface roughness of the substrate and the sensing characteristics of FBG has not yet been clearly revealed.

This paper aims to improve the measurement accuracy and stability of FBG sensors and focuses on conducting experimental research on the influence of the surface roughness of the packaging substrate on the sensing performance of FBG. The study selects an equal-strength metal cantilever beam as the packaging substrate and builds a gradient-shaped roughness morphology on the beam surface through sandblasting technology. By using a comparison experiment system, the influence laws of different surface roughnesses on the strain transmission efficiency, linearity, and repeatability of the FBG sensor are analyzed. Finally, the optimal roughness range of the FBG packaging substrate surface is determined, providing a theoretical basis and technical support for the packaging design of high-performance FBG sensors.

## 2. Experimental Principle

**Figure 1:**
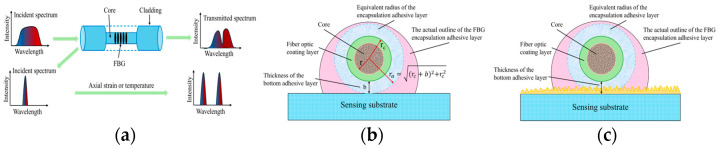
(**a**) FBG sensing principle; (**b**) schematic diagram of encapsulation with smooth sensor substrate surface; (**c**) schematic diagram of sensor matrix encapsulation under surface roughness conditions.

FBG sensors operate on the principle of Bragg diffraction. When light interacts with the grating—a structure characterized by periodic variations in the refractive index along the fiber core—light of a specific wavelength will be reflected back, as illustrated in Figure 1a [26].

According to the FBG sensing principle diagram in Figure 1a, combined with the theory of the optical fiber coupling model [8,27], the central wavelength *λ_B_* of the reflected signal is related to the refractive index variation period and the effective refractive index of the grating, and is expressed as [28]:

(1) $$\lambda_{B}=2n_{\mathrm{eff}}\Lambda$$

Here, *λ_B_* represents the central wavelength of the FBG, n_eff_ is the effective refractive index of the optical fiber, and Λ is the grating period.

This Equation reflects the relationship among the central wavelength of the grating, the grating period, and the effective refractive index, and is the theoretical basis for fiber optic grating sensing. This formula indicates that the central wavelength of the FBG is affected by the period. When the FBG is influenced by external factors and changes, its period *Λ* will also change, thereby causing the central wavelength of the FBG to drift. The influence of strain and temperature changes on the drift of the FBG’s central wavelength can be expressed as [29]:

(2) $$\frac{\Delta \lambda }{\lambda }=\left(\alpha_{f}+\xi \right)\times \Delta T+\left(1-P_{e}\right)\times \Delta \varepsilon$$

In the equation, *λ* is the initial wavelength of the FBG, Δ*T* is the temperature change, and ∆ε is the strain change; Δ*λ* is the central wavelength change in the fiber Bragg grating; α_f_ is the thermal expansion coefficient, *ξ* is the thermal optical coefficient, and *Pe* is the photoelastic coefficient [30].

During the FBG encapsulation process, Figure 1b,c shows cross-sectional views of the encapsulated FBG under different surface conditions of the sensing substrate. The FBG is combined with the substrate through the packaging adhesive layer. The strain change Δε of the FBG is essentially the result of the strain transfer between the substrate and the packaging adhesive layer. The efficiency of strain transfer depends on the interface bonding state between the FBG and the substrate, as well as the packaging adhesive layer. Interface detachment is one of the main failure forms of the packaging adhesive layer–substrate system.

The strain energy release rate *G* is a key indicator for characterizing the debonding driving force. When G reaches the critical value *G*_cr_, debonding begins to occur. *G*_cr_ depends on interface parameters (such as phase angle ψ’), and in combination with the influence of the diffusion substance concentration in the adhesive layer, the rate of change $G^{\prime }=\partial G/\partial t$ of the strain energy release rate over time can be described by the following relationship:

(3) $$G^{\prime }=k_{1}\cdot \cap \cdot M_{c}$$

$\cap =\sigma_{dc}^{\prime }/\sigma_{cr}$ depends on the time-dependent characteristics of diffusion-induced stress, $Mc=c_{kc}^{\prime }h_{\alpha }/2$ denotes the applied moment acting on the adhesive layer with thickness *h_α_*, and $k_{1}=0.5\sqrt{{\left(1+v\right)}^{2}/(1+v)+(1-v)}$ is a constant related to Poisson’s ratio v of the adhesive layer.

When condition $\cap \geq \cap_{\mathrm{cr}}$ is satisfied, G’ can be related to the intrinsic toughness of the interface through the dimensionless modal mixing function *j* [31], where $G^{\prime }\approx \Gamma_{IC}$ holds and $\Gamma_{IC}\approx G_{cr}$. The modal mixing function j is determined by the interface roughness *λ* and the elastic mismatch parameter:

(4) $$j(\lambda )=\frac{1}{1+k_{2}\cdot \omega \cdot \lambda }$$

where $k_{2}=\sqrt{{\left[0.2(1+v)+(1-v^{2})\right]}^{-1}}$ is a constant associated with the Poisson ratio, ω depends on the Dundur elastic mismatch parameter $\chi =E_{1}-E_{2}/E_{1}+E_{2}$ (where E_1_ and E_2_ denote the elastic moduli of the adhesive layer and substrate, respectively) [32], and $\lambda =R_{a}^{2}/L_{a}$ (where R_a_ denotes the average roughness amplitude and L_a_ denotes the average roughness wavelength).

The partial differential equation (PDE) for the interfacial debonding driving force F is:

(5) $$\frac{\partial F}{\partial t}=k_{3}\cdot c_{kc}^{\prime 2}+k_{4}\cdot \cap (h_{\alpha })-B\cdot j(\lambda )\cdot F$$

$k3=6(1-v^{2})/E_{c}$ (E_c_ denotes the elastic modulus of the adhesive layer), and $k_{4}=0.25(1+v)k_{2}$, $B=\Gamma_{IC}/E_{c}\lambda$. This equation indicates that the debonding driving force F is jointly influenced by the concentration of the diffusing substance in the adhesive layer, the interfacial roughness λ, and the coating thickness *h_α_*.

The surface roughness λ solely modifies the amplitude of F through j(λ). At time $\lambda >\lambda_{cr}$, *j*(*λ*) increases, *F* decreases, and the interface bonding tends towards stability; at time $\lambda <\lambda_{cr}$, *j*(*λ*) decreases, F increases, and the risk of debonding rises.

The coating thickness *h_α_* will affect the time discretization of the debonding driving force *F*.

The interfacial coupling state between an FBG and the substrate directly determines the strain transfer efficiency. When the interfacial debonding driving force F does not reach the critical level, the adhesive layer maintains a tight bond with the substrate, and the strain of the substrate can be effectively transferred to the FBG through the interface. In this case, the strain Δ*ε* sensed by the FBG and the actual strain Δ*ε_sub_* of the substrate satisfy a linear transfer relationship:

(6) $$\Delta \varepsilon =\eta \cdot \Delta \varepsilon_{sub}$$

*η* denotes the strain transfer efficiency with the range of *η*∈ (0, 1], whose value is determined by the interfacial bonding strength. The tighter the interfacial bonding, the closer *η* is to 1, and the more accurately the FBG central wavelength shift Δ*λ* can reflect the variation in substrate strain.

When the debonding driving force F increases and approaches the critical value, micro-debonding or debonding propagation initiates at the interface, whereupon the strain transfer capacity of the interface decreases significantly. From the perspective of mechanical mechanisms, interfacial debonding disrupts the stress transfer path between the FBG and the substrate, leading to stress relaxation in local regions, such that the actual strain Δ*ε* borne by the FBG satisfies:

(7) $$\Delta \varepsilon =\eta (F)\cdot \Delta \varepsilon_{sub}$$

*η*(F) denotes the strain transfer efficiency function dependent upon the debonding driving force, and according to dη/dF<0, the greater the debonding driving force F, the smaller η(F) becomes. When F reaches the critical value F_cr_, the interface becomes completely detached, $\eta (F_{cr})=0$. The FBG no longer senses the substrate strain, and its strain Δε≈0.

Combining the wavelength drift formula for FBGs, the wavelength drift characteristics when the debonding driving force is excessive are as follows:

(8) $$\Delta \lambda =\lambda_{0}[(\alpha_{f}+\xi )\Delta T+P_{e}\cdot \eta (F)\cdot \Delta \varepsilon_{sub}]$$

Since temperature variations Δ*T* can typically be monitored and eliminated independently via temperature-compensated FBGs, under temperature-stable conditions (Δ*T* = 0), wavelength drift is solely determined by strain transmission:

(9) $$\Delta \lambda =\lambda_{0}P_{e}\cdot \eta (F)\cdot \Delta \varepsilon_{sub}$$

An increase in the interfacial debonding driving force F leads to a decrease in the strain transfer efficiency *η*(F), thereby reducing the center wavelength drift Δ*λ* of the FBG. When *F* becomes too large and causes severe off-stretching, *η*(*F*) approaches 0, and Δ*λ* approaches the drift reference value corresponding to the initial wavelength. At this point, the FBG is unable to effectively reflect the actual strain state of the substrate.

## 3. Materials and Methods

**Figure 2:**
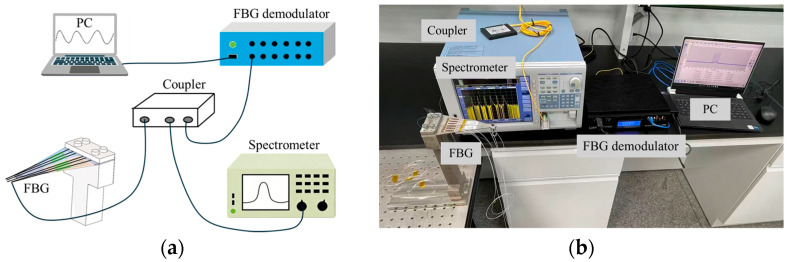
(**a**) Schematic diagram of the overall experimental setup; (**b**) overall experimental setup.

In this study, equal-strength metal beams were selected as the elastic elements. When an equal-strength beam undergoes bending deformation, the strain distribution on the beam surface is uniform and consistent. This ensures that the tensile or compressive strains applied to the fiber Bragg grating (FBG) attached to the surface of the beam are the same. Thus, the FBG will not exhibit the chirp phenomenon due to uneven local stress. The FBG demodulator, spectrum analyzer, and FBG encapsulated on the equal-strength beam are connected using a coupler to form a complete experimental system. The overall experimental setup is shown in Figure 2.

To systematically investigate the influence of the surface roughness of the sensing substrate on the packaging effect and performance of FBG, this study selected eight different particle sizes of sandblasting abrasives to treat the substrate surface and conducted experiments. The abrasive particle sizes were 16#, 36#, 60#, 100#, 150#, 220#, 320#, and 1000#.

The design scheme of a double-zone single-beam layout is adopted to eliminate the interference of individual differences in the base material on the test data and ensure the singleness of the test variables. The two different particle sizes of abrasives are subjected to sandblasting treatment in two independent zones of the same strength beam. The specific grouping scheme is as follows: The two zones of the base material of the first beam are respectively treated with 16# and 36# abrasives for sandblasting; the corresponding area of the second base material is treated with 60# and 100# abrasives; the double zone of the third base material implements sandblasting modification with 150# and 220# abrasives; and the fourth base material is completed with 320# and 1000# abrasives for sandblasting operation.

**Figure 3:**
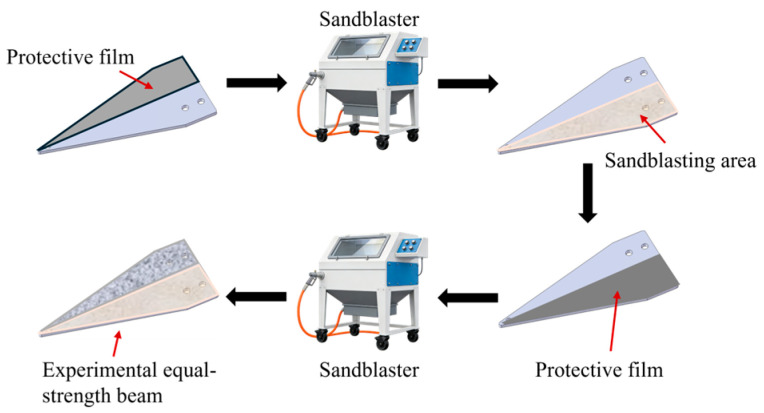
Sandblasting processing procedure.

**Figure 4:**
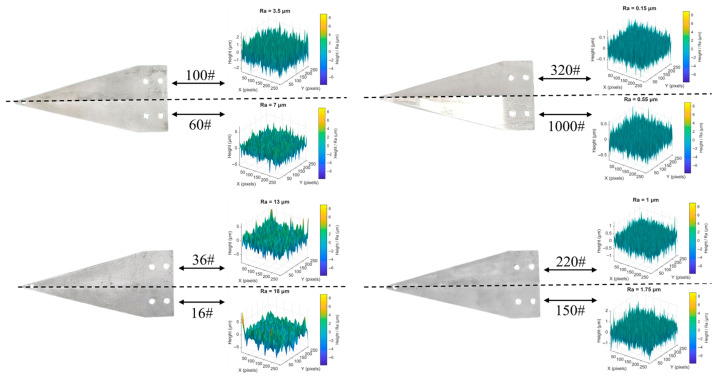
Simulation of equal-strength beams after sandblasting treatment and their surface roughness.

Before sandblasting, a plastic film is first fully wrapped around one side of the equal-strength beam, and then the plastic film is fixed with tape. This operation method can effectively prevent residual adhesive residues from the non-treated side of the beam from remaining after single-sided sandblasting and prevent the adhesive residues from causing interference to the subsequent processing. After one side of the beam was sandblasted, a plastic film and tape are used to seal and protect the area that had undergone sandblasting. Then, for the other side area, different particle sizes of abrasive materials are used for sandblasting. Surface roughness of the substrate is controlled through the precise selection of sandblasting equipment and abrasives. The sandblasting manufacturer delivers the substrate only after confirming that the surface roughness meets specifications following sandblasting treatment. Figure 3 shows the sandblasting process of the equal-strength beam. Figure 4 shows the equal-strength beam after sandblasting and its corresponding roughness simulation.

**Figure 5:**
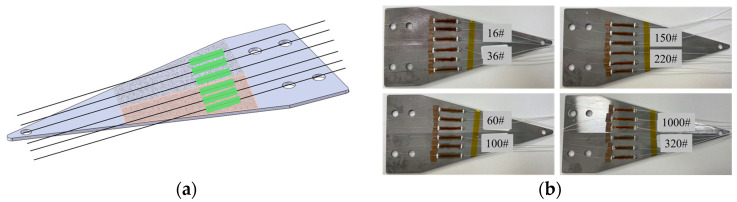
(**a**) Schematic of FBG packaging; (**b**) FBG packaging diagram.

On the sandblasted equal-strength beam, three FBGs are bonded and packaged in each region with distinct surface roughness to monitor the surface strain variation in the substrate and eliminate random errors in the experiment. The packaging process is shown in Figure 5a. The specific steps are as follows: First, the 6 fiber Bragg gratings required for each equal-strength beam are removed from the coating layer of the grating area and fixed at the preset position on the beam. Then, the curing adhesive is evenly applied to the grating area of the FBG for full adhesion packaging. Compared with the two-point encapsulation, the full encapsulation in this method ensures that the adhesive layer wraps around the FBGs when they are subjected to axial pressure, providing certain protection for the FBGs and preventing them from easily breaking. The fiber curing adhesive selected is EPO-TEK 353ND, which is commonly used in the preparation process of fiber Bragg grating sensors. The experiment selected this curing adhesive to make the results more universal. The full encapsulation and pasting length of each group of 6 FBGs remained consistent, with a length of 20 mm. During the gluing process, the adhesive layer thickness is approximately 1 mm. After heating and curing on the heating platform and natural cooling, the encapsulation of each group of 6 fiber Bragg gratings (a total of 24 FBGs) was completed. Figure 5b shows the final effect after the overall encapsulation of the FBGs.

## 4. Experiments and Analysis of Results

### 4.1. Load Test

A load test was conducted on the fiber Bragg grating (FBG) encapsulated on an equal-strength beam under room temperature conditions. Prior to the experiment, the fixed end of the beam was securely mounted onto the base of the experimental platform to ensure the beam remained parallel to the platform surface. During testing, the free end of the beam was positioned vertically at a 90° angle relative to the platform. Starting from the no-load initial state, weights were suspended sequentially in increments of 5 N until reaching a maximum load of 75 N. Each weight was held in place for 30 s, and the next weight was applied only after the wavelength drift had stabilized.

**Figure 6:**
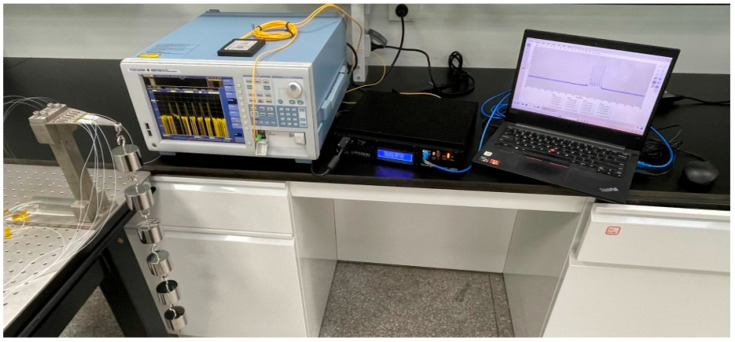
Load testing experiment.

Throughout the experiment, the wavelength drift of the FBG was monitored and recorded in real time using a spectral analyzer (Yokogawa Electric Corporation, Tokyo, Japan) and a fiber grating demodulator (Luna Innovations, Roanoke, VA, USA). The demodulator featured a wavelength resolution of 0.1 pm and an operating bandwidth spanning 1520–1600 nm, while the wavelength data output by the FBG was analyzed and documented with an accuracy of 2 pm. All experiments in this article were conducted under constant temperature conditions. The overall experimental setup is illustrated in Figure 6, and Table 1 presents the initial center wavelengths of the 24 FBGs utilized in this study. All FBGs used in the experiment were sourced from the same manufacturer and batch.

**Figure 7:**
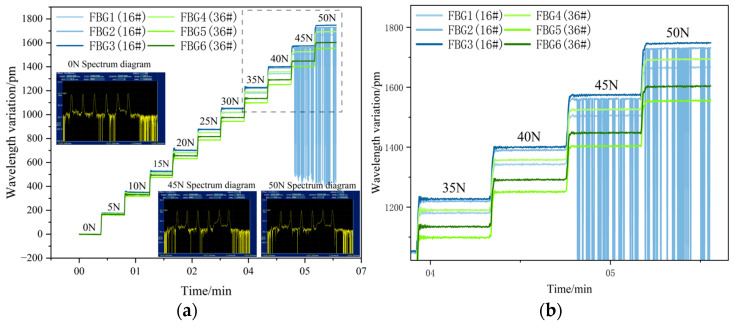
(**a**) Time course diagram of wavelength drift of FBG1 to FBG6 output wavelengths; (**b**) the wavelength drift of FBG1 to FBG6 output at 35 to 50 N.

The time history of the wavelength drift of FBG1 to FBG6 attached to the No. 1 equal-strength beam during the experiment is shown in Figure 7. When the load was applied to 45 N and 50 N, FBG2 showed a significant chirp phenomenon, which made it impossible to conduct accurate measurements for subsequent loads and repetitive experiments. The other FBGs did not show obvious chirp phenomena at the demodulator output end. However, using a spectral analyzer, it was found that the spectra of the remaining FBGs all had varying degrees of chirp distortion. By magnifying the data in the 35 N to 50 N load range for analysis, it was found that when the load reached 45 N, the strain transfer characteristics between the substrate and FBG had lost their highly sensitive response capability. Therefore, the surface roughness of the sensing substrate was too large. As the applied load gradually increased, the debonding driving force F increased, and the interface-coupling ability between the sensing substrate and the adhesive layer decreased, resulting in a reduction in strain transfer efficiency and the failure of strain transfer between FBG and the sensing substrate. At this point, FBG1 to FBG6 could no longer reflect the true strain state of the sensing substrate, and chirp phenomena occurred.

**Figure 8:**
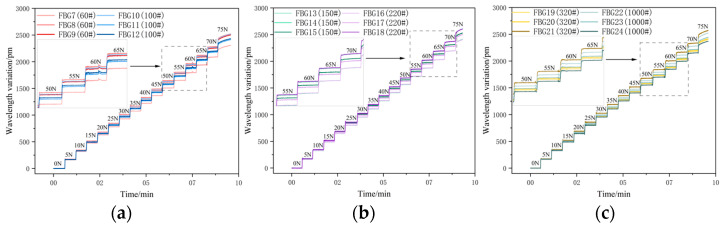
(**a**) Time course diagram of wavelength drift of FBG7 to FBG12 output; (**b**) time course diagram of wavelength drift of FBG13 to FBG18 output; (**c**) time course diagram of wavelength drift of FBG19 to FBG24 output.

Within the low-load range of 0 to 50 N, as shown in Figure 8a, the wavelength shifts in FBG7 to FBG12 increase linearly with the load, indicating that the interface bonding state is stable at this stage and the strain transfer efficiency remains at a high level. When the load rises to 55 N, the strain transfer rate of FBG significantly decreases, the curve slope changes significantly, the interface-coupling ability declines, and there is a loss in the strain transfer process.

In Figure 8b, FBG13 to FBG18 maintained a stable linear response within the load range of 0 to 60 N. The interface-coupling capability between the sensing substrate and the FBG remained in a stable state within 60 N, enabling strain transmission under higher loads. When the load increased to 65 N, the interface-coupling capability decreased, the detachment driving force F increased, the strain transmission efficiency declined, and the linearity of the wavelength drift began to deteriorate.

In Figure 8c, FBG19 to FBG24 exhibit a characteristic of decreased strain transfer efficiency under a 55 N load. Under high load conditions, the strain transfer efficiency is weaker than that of the FBGs encapsulated on beams No. 2 and No. 3. The interface-coupling capability of this beam body is relatively weak, and it cannot effectively transfer strain under high load conditions. However, its interface-coupling capability is better than that of beam No. 1.

When the load reaches 75 N, FBG7 to FBG24 reach the debonding critical point, and the interface-coupling ability is weak. At this time, the strain transmission completely fails, and the wavelength drift of FBG cannot accurately reflect the true strain state of the matrix.

The load test results show that FBG1 to FBG6 all exhibited chirp distortion in the spectrum analyzer when the load was increased to 45 N (1.143 × 10^−3^/ε). This phenomenon indicates that when the surface roughness of the substrate is too large, the interface between the FBG and the substrate is prone to form local stress concentration, which disrupts the axial strain distribution of the FBG, reduces the interface-coupling ability, and causes the FBG to exhibit chirp distortion and strain transmission failure.

### 4.2. Repetitive Experiment

**Figure 9:**
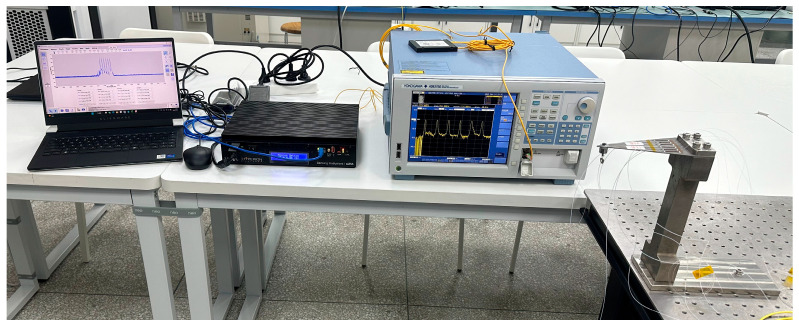
Tensile test experiment.

To explore the sensing stability and repeatability of fiber Bragg grating (FBG) under cyclic loading, tensile cyclic experiments were conducted at room temperature. The equal-strength beam was fixed on the experimental base, with the top of the beam serving as the force application point. Axial loads were applied by gradually adding weights. The unloaded state was set as the initial reference for wavelength collection. The load was increased in steps of 1 N up to 15 N. After each load step was applied, the load was held stable for 30 s. Once the output wavelength of the FBG stabilized, data collection was carried out. Then, the load was unloaded in the same steps back to the unloaded state, completing one full tensile cycle experiment. To ensure the reliability of the experimental data, the loading–unloading cycle was repeated three times. The overall layout of the experimental system is shown in Figure 9.

During the data analysis process, the load (N) applied to the beam is converted into strain (ε) using the equal-strength beam strain formula, as follows:

(10) $$\varepsilon =\frac{6L}{Ebh^{2}}\cdot F$$

**Figure 10:**
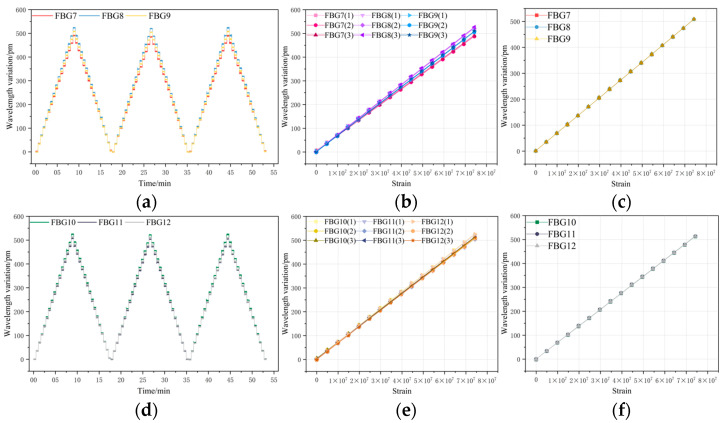
(**a**) Time–history curves of output wavelength drift: FBG7–FBG9; (**b**) data graph of 3 instances of stretching cycle test; (**c**) wavelength drift averaged over three test cycles for FBG7–FBG9; (**d**) time–history curves of output wavelength drift: FBG10–FBG12; (**e**) data graph of 3 instances of stretching cycle test; (**f**) wavelength drift averaged over three test cycles for FBG10–FBG12.

**Figure 11:**
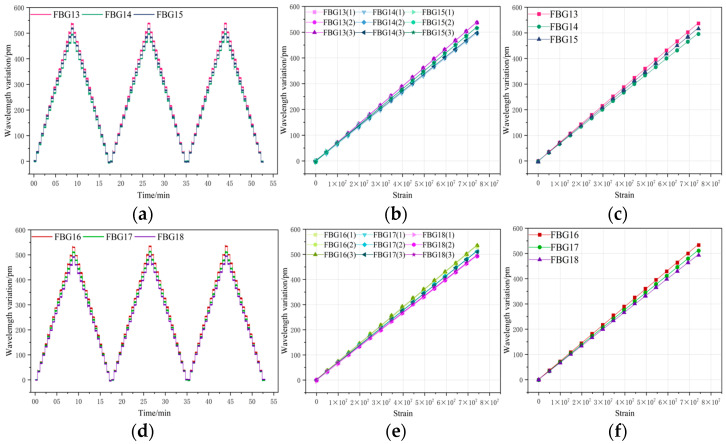
(**a**) Time–history curves of output wavelength drift: FBG13–FBG15; (**b**) data graph of 3 instances of stretching cycle test; (**c**) wavelength drift averaged over three test cycles for FBG13–FBG15; (**d**) time–history curves of output wavelength drift: FBG16–FBG18; (**e**) Data graph of 3 instances of stretching cycle test; (**f**) wavelength drift averaged over three test cycles for FBG16–FBG18.

**Figure 12:**
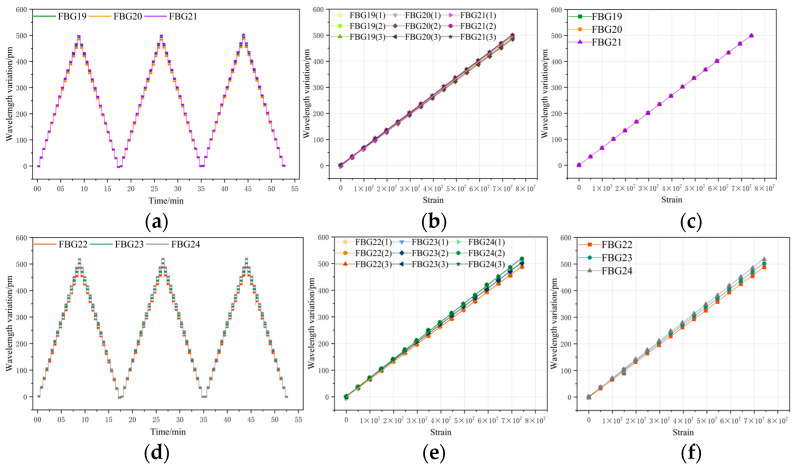
(**a**) Time–history curves of output wavelength drift: FBG19–FBG21; (**b**) Data graph of 3 instances of stretching cycle test; (**c**) wavelength drift averaged over three test cycles for FBG19–FBG21; (**d**) time–history curves of output wavelength drift: FBG22–FBG24; (**e**) data graph of 3 instances of stretching cycle test; (**f**) wavelength drift averaged over three test cycles for FBG22–FBG24.

Figure 10, Figure 11 and Figure 12 present the overall experimental data of the repeated tensile tests for FBGs packaged on six groups of sensing substrates with distinct surface roughness. In each group of Figures, (a) and (d) show the time–history curves of the wavelength shift output by the FBGs, while (b) and (e) are the plots of three cyclic tensile test data for each FBG extracted from the real-time curves in (a) and (d), illustrating the wavelength shift in FBGs during loading and unloading at each strain point across the three cyclic tests. To ensure the accuracy of the subsequent experimental processing results, the data presented in (c) and (f) are the arithmetic mean values calculated from the three experimental datasets of each FBG in (b) and (e), which yield the average wavelength shift output by FBG7 to FBG24 throughout the entire experimental process. Excluding FBG1 to FBG6, which had undergone chirping during the load tests and were thus not measured, the data in the Figures indicate that the wavelength shifts in FBG7 to FBG24 are essentially consistent in the repeated tests, all exhibiting excellent repeatability for measurement.

**Figure 13:**
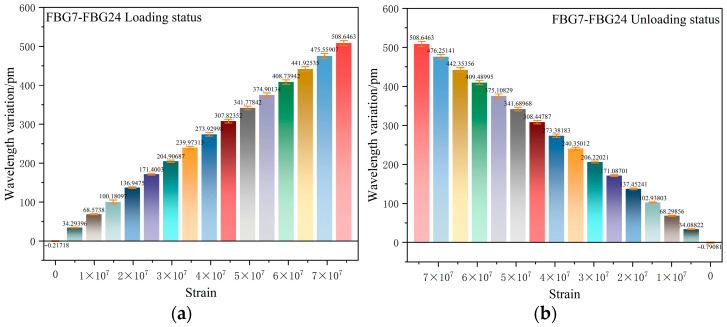
(**a**) FBG7-FBG24 loading status error analysis; (**b**) FBG7-FBG24 unloading status error analysis.

From Figure 13a, the average relative error of FBG7–FBG24 during loading is approximately 2.1–3.5%. In (b), the average relative error during unloading is approximately 2.0–3.4%, with the maximum relative error not exceeding 3.5%. This is well below the acceptable threshold in typical experiments.

**Figure 14:**
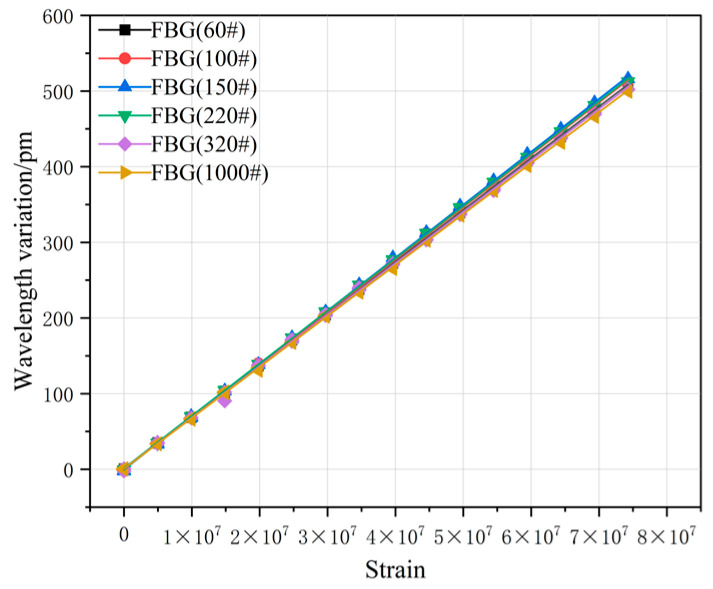
Linearly fitted data graph of the average value of FBG.

To further clarify the influence law of different surface roughness regions on the strain-sensing characteristics of fiber Bragg gratings (FBGs), for the test curve data of the three FBGs corresponding to each roughness region in Figure 10c,f, Figure 11c,f and Figure 12c,f, the arithmetic mean method was adopted to fuse the three strain–wavelength drift curves within the same region to eliminate the random errors in the single grating test process. On this basis, the mean data of each roughness region were linearly fitted to construct the test curve data graph between strain and wavelength drift. The FBG wavelength change characteristic curves after arithmetic averaging processing in different roughness regions are shown in Figure 14, and the corresponding linear fitting equations and detailed fitting parameters are listed in Table 2.

By conducting a comprehensive analysis of the experimental data in Figure 10, Figure 11, Figure 12, Figure 13 and Figure 14, the correlation between the wavelength drift Δλ of the fiber Bragg grating (FBG) and the external strain ε can be clearly determined. Based on the linear fitting equations and parameters of the FBG in each roughness area in Table 2, it can be concluded that the FBG sensing performance in the 150# grit sandblasting treatment area is the best. The linear fitting equation obtained by taking the arithmetic mean of the three FBG test curves in this area is y = −0.26237 + 6.99994 × 10^−6^x, and the strain response capability is as high as 6.99994 × 10^−6^ pm/ε, with a linear correlation coefficient reaching 0.99994. This result indicates that the strain transfer efficiency between the surface roughness of the sensing substrate treated with 150# grit and the FBG is the highest, with stable response characteristics and excellent linearity.

In contrast, during the experimental testing of the FBG in the 320# and 1000# abrasive blasting treatment areas, as the load increased and was under long-term testing conditions, the detachment driving force F gradually increased, the interface-coupling ability decreased, and the strain transmission efficiency significantly reduced. In the repeatability experiments, it exhibited the poorest sensing performance, with poor repeatability of the wavelength drift amount, and a significant reduction in the linearity of the strain–wavelength change response. Thus, it can be seen that when the surface roughness of the substrate is too small, the interface-coupling ability between the FBG and the sensing substrate is poor, resulting in insufficient interface bonding strength of the packaging structure. During the sensor preparation and packaging process, if the roughness of the sensing substrate surface is in this range, it not only significantly reduces the sensor’s sensitivity but also seriously affects its long-term service stability and service life.

## 5. Conclusions

This study used equal-strength beams as the base specimens and controlled the surface sandblasting process parameters to prepare different roughness levels of the base interface. It systematically explored the influence laws of the base surface roughness on the packaging effect and sensing performance of fiber Bragg gratings (FBGs), and finally determined the optimal range of base surface roughness suitable for the high-precision packaging of FBGs. The main research conclusions are as follows:

(1) The static load test results show that the FBGs encapsulated in the 16# and 36# abrasive sandblasting treatment areas all exhibited obvious chirp phenomena when the load was applied to 45 N, and the experiments could not continue.
(2) The repeatability test shows that the FBGs in the 320# and 1000# abrasive sandblasting treatment areas had the poorest sensing performance. After three cycles, the wavelength drift of the FBGs in this type of area significantly decreased in repeatability, and the strain response coefficient was the worst.
(3) From the data of multiple groups of load and repeatability tests, it can be concluded that the base surface treated with 150# abrasive sandblasting is the best choice for FBG packaging, and all the sensing performance indicators of this type of base surface reach the optimal level.
(4) When the substrate surface roughness is low, strain transmission differences are minimal and do not cause chirping phenomena, affecting only sensitivity. When substrate surface roughness is excessive, strain transmission differences become too large, causing chirping in the FBG and rendering measurement sensing impossible.

This study explored the correlation between the base surface roughness and the packaging performance of FBGs. The obtained conclusions can provide key technical support for the optimization design of the packaging interface in the development of fiber Bragg grating sensors and have important practical guiding significance for improving the core performance of the sensors.

## Figures and Tables

**Table 1:** Initial central wavelength of FBG.

	FBG Wavelength/nm		FBG Wavelength/nm
FBG1	1537	FBG13	1555
FBG2	1540	FBG14	1558
FBG3	1543	FBG15	1561
FBG4	1528	FBG16	1546
FBG5	1531	FBG17	1549
FBG6	1534	FBG18	1552
FBG7	1537	FBG19	1564
FBG8	1540	FBG20	1567
FBG9	1543	FBG21	1570
FBG10	1528	FBG22	1573
FBG11	1531	FBG23	1576
FBG12	1534	FBG24	1579

**Table 2:** Linear fitting expression for the mean value of FBG.

	Curve-Fitting Equation	Linearity R	Strain Response (pm/ε)
FBG (60#)	y = 1.4366 + 6.83093 × 10^−6^x	0.99994	6.83093 × 10^−6^
FBG (100#)	y = 0.50294 + 6.91961 × 10^−6^x	0.99991	6.91961 × 10^−6^
FBG (150#)	y = −0.26237 + 6.99994 × 10^−6^x	0.99994	6.99994 × 10^−6^
FBG (220#)	y = 1.40963 + 6.9289 × 10^−6^x	0.99992	6.9289 × 10^−6^
FBG (320#)	y = 0.74463 + 6.79256 × 10^−6^x	0.99989	6.79256 × 10^−6^
FBG (1000#)	y = 0.63848 + 6.73683 × 10^−6^x	0.99971	6.73683 × 10^−6^

## Data Availability

Dataset available on request from the authors.
